# *N*-(2-Methyl­phenyl­sulfon­yl)acetamide

**DOI:** 10.1107/S1600536811014218

**Published:** 2011-04-22

**Authors:** K. Shakuntala, Sabine Foro, B. Thimme Gowda

**Affiliations:** a Department of Chemistry, Mangalore University, Mangalagangotri 574 199, Mangalore, India; b Institute of Materials Science, Darmstadt University of Technology, Petersenstrasse 23, D-64287 Darmstadt, Germany

## Abstract

In the mol­ecular structure of the title compound, C_9_H_11_NO_3_S, the N—H and C=O bonds are *anti* to each other, while the amide H atom is *syn* with respect to the *ortho*-methyl group in the benzene ring. The C—S—N—C torsion angle is −58.2 (2)°, indicating a twist in the mol­ecule. In the crystal, N—H⋯O hydrogen bonds link the mol­ecules into chains along the *c* axis.

## Related literature

For the sulfanilamide moiety in sulfonamide drugs, see: Maren (1976 ▶). For hydrogen bonding modes of sulfonamides, see: Adsmond & Grant (2001 ▶). For our study of the effect of substituents on the structures of *N*-(ar­yl)-amides, see: Gowda *et al.* (2004 ▶). For background to the structures of *N*-(substituted phenyl­sulfon­yl)-substituted-amides, see: Gowda *et al.* (2010 ▶); Shakuntala *et al.* (2011 ▶) and for the oxidative strengths of *N*-chloro, *N*-aryl­sulfonamides, see: Gowda & Kumar (2003 ▶).
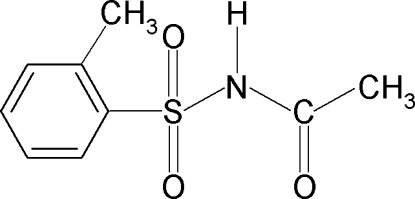


## Experimental

### 

#### Crystal data

C_9_H_11_NO_3_S
*M* *_r_* = 213.25
Tetragonal, 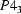

*a* = 7.9804 (5) Å
*c* = 16.749 (1) Å
*V* = 1066.69 (11) Å^3^
*Z* = 4
Mo *K*α radiation
μ = 0.29 mm^−1^
*T* = 293 K
0.40 × 0.18 × 0.12 mm

#### Data collection

Oxford Diffraction Xcalibur diffractometer with a Sapphire CCD detector
Absorption correction: multi-scan (*CrysAlis RED*; Oxford Diffraction, 2009 ▶) *T*_min_ = 0.895, *T*_max_ = 0.967
4245 measured reflections
1944 independent reflections
1690 reflections with *I* > 2σ(*I*)
*R*_int_ = 0.016

#### Refinement

*R*[*F* ^2^ > 2σ(*F* ^2^)] = 0.035
*wR*(*F* ^2^) = 0.086
*S* = 1.08
1944 reflections
132 parameters
2 restraints
H atoms treated by a mixture of independent and constrained refinement
Δρ_max_ = 0.14 e Å^−3^
Δρ_min_ = −0.17 e Å^−3^
Absolute structure: Flack (1983 ▶), 824 Friedel pairs
Flack parameter: 0.03 (9)

### 

Data collection: *CrysAlis CCD* (Oxford Diffraction, 2009 ▶); cell refinement: *CrysAlis RED* (Oxford Diffraction, 2009 ▶); data reduction: *CrysAlis RED*; program(s) used to solve structure: *SHELXS97* (Sheldrick, 2008 ▶); program(s) used to refine structure: *SHELXL97* (Sheldrick, 2008 ▶); molecular graphics: *PLATON* (Spek, 2009 ▶); software used to prepare material for publication: *SHELXL97*.

## Supplementary Material

Crystal structure: contains datablocks I, global. DOI: 10.1107/S1600536811014218/tk2737sup1.cif

Structure factors: contains datablocks I. DOI: 10.1107/S1600536811014218/tk2737Isup2.hkl

Additional supplementary materials:  crystallographic information; 3D view; checkCIF report

## Figures and Tables

**Table 1:** Hydrogen-bond geometry (Å, °)

*D*—H⋯*A*	*D*—H	H⋯*A*	*D*⋯*A*	*D*—H⋯*A*
N1—H1*N*⋯O3^i^	0.86 (2)	1.95 (2)	2.770 (3)	162 (3)

Symmetry code: (i) 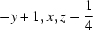.

**Fig. 1.**
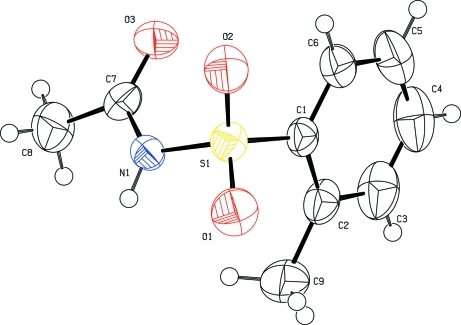
Molecular structure of (I), showing the atom-labelling scheme. Displacement ellipsoids are drawn at the 50% probability level.

**Fig. 2.**
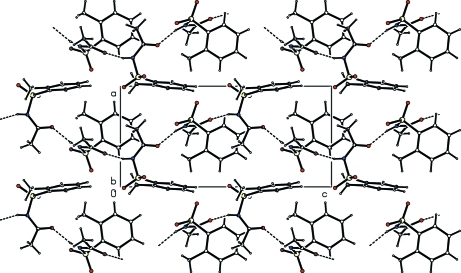
View of the crystal packing in (I). Hydrogen bonds are shown as dashed lines.

## supplementary crystallographic information

### Comment

The structures of sulfonamide drugs contain the sulfanilamide moiety (Maren, 1976). The hydrogen bonding preferences of sulfonamides has been investigated (Adsmond & Grant, 2001). The nature and position of the substituents play a significant role on their crystal structures and other aspects of *N*-(aryl)-amides and *N*-(aryl)-sulfonamides (Gowda *et al.*, 2003, 2004; Shakuntala *et al.*, 2011). As a part of a study of the effects of substituents on the structures of this class of compounds, the structure of *N*-(2-methylphenylsulfonyl)-acetamide (I) has been determined (Fig. 1). The conformation of the N—C bond in the C—SO_2_—NH—C(O) segment has *gauche* torsions with respect to the S═O bonds, the torsion angles being C7—N1—S1—O2 = 57.5 (3) ° and C7—N1—S1—O1 = -174.5 (2) °.

The N—H and C═O bonds are *anti* to each other, similar to that observed in *N*-(phenylsulfonyl)-acetamide (II) (Gowda *et al.*, 2010) and *N*-(2-chlorophenylsulfonyl)-acetamide (III) (Shakuntala *et al.*, 2011). Further, the conformation of the amide-H atom is *syn* to the *ortho*-methyl group in the benzene ring, similar to that observed between the amide-H atom and the *ortho*-chloro group in (III). The molecule of (I) is bent at the *S*-atom with a C—S—N—C torsion angle of -58.2 (2) °, compared to the values of -58.8 (4) ° in (II), and -71.7 (3) and 61.2 (3) ° in the two independent molecules of (III).

In the crystal structure, intermolecular N—H···O hydrogen bonds (Table 1) link the molecules into chains along the *c* axis; part of the crystal structure is shown in Fig. 2.

### Experimental

The title compound was prepared by refluxing 2-methylbenzenesulfonamide (0.10 mole) with an excess of acetyl chloride (0.20 mole) for one hour on a water bath. The reaction mixture was cooled and poured into ice cold water. The resulting solid was separated, washed thoroughly with water and dissolved in warm dilute sodium hydrogen carbonate solution. The title compound was re-precipitated by acidifying the filtered solution with glacial acetic acid. It was filtered, dried and recrystallized from ethanol. Colourless rods of the title compound were obtained from a slow evaporation of its ethanol solution.

### Refinement

The H atom of the NH group was located in a difference map and later restrained to the distance N—H = 0.86±0.02 Å. The other H atoms were positioned with idealized geometry using a riding model with the aromatic C—H distance = 0.93 Å and methyl C—H = 0.96 Å. All H atoms were refined with isotropic displacement parameters set to 1.2 times of the *U*_eq_ of the parent atom.

### Crystal data

**Table:**

C_9_H_11_NO_3_S	*D*_x_ = 1.328 Mg m^−^^3^
*M**_r_* = 213.25	Mo *K*α radiation, λ = 0.71073 Å
Tetragonal, *P*4_3_	Cell parameters from 1894 reflections
Hall symbol: P 4cw	θ = 2.5–27.8°
*a* = 7.9804 (5) Å	µ = 0.29 mm^−^^1^
*c* = 16.749 (1) Å	*T* = 293 K
*V* = 1066.69 (11) Å^3^	Prism, colourless
*Z* = 4	0.40 × 0.18 × 0.12 mm
*F*(000) = 448	

### Data collection

**Table:**

Oxford Diffraction Xcalibur diffractometer with a Sapphire CCD detector	1944 independent reflections
Radiation source: fine-focus sealed tube	1690 reflections with *I* > 2σ(*I*)
graphite	*R*_int_ = 0.016
Rotation method data acquisition using ω and φ scans	θ_max_ = 26.4°, θ_min_ = 2.6°
Absorption correction: multi-scan (*CrysAlis RED*; Oxford Diffraction, 2009)	*h* = −9→5
*T*_min_ = 0.895, *T*_max_ = 0.967	*k* = −6→9
4245 measured reflections	*l* = −17→20

### Refinement

**Table:**

Refinement on *F*^2^	Secondary atom site location: difference Fourier map
Least-squares matrix: full	Hydrogen site location: inferred from neighbouring sites
*R*[*F*^2^ > 2σ(*F*^2^)] = 0.035	H atoms treated by a mixture of independent and constrained refinement
*wR*(*F*^2^) = 0.086	*w* = 1/[σ^2^(*F*_o_^2^) + (0.0453*P*)^2^ + 0.1227*P*] where *P* = (*F*_o_^2^ + 2*F*_c_^2^)/3
*S* = 1.08	(Δ/σ)_max_ = 0.016
1944 reflections	Δρ_max_ = 0.14 e Å^−^^3^
132 parameters	Δρ_min_ = −0.17 e Å^−^^3^
2 restraints	Absolute structure: Flack (1983), 824 Friedel pairs
Primary atom site location: structure-invariant direct methods	Flack parameter: 0.03 (9)

### Special details

**Table:**

Experimental. CrysAlis RED (Oxford Diffraction, 2009) Empirical absorption correction using spherical harmonics, implemented in SCALE3 ABSPACK scaling algorithm.
Geometry. All e.s.d.'s (except the e.s.d. in the dihedral angle between two l.s. planes) are estimated using the full covariance matrix. The cell e.s.d.'s are taken into account individually in the estimation of e.s.d.'s in distances, angles and torsion angles; correlations between e.s.d.'s in cell parameters are only used when they are defined by crystal symmetry. An approximate (isotropic) treatment of cell e.s.d.'s is used for estimating e.s.d.'s involving l.s. planes.
Refinement. Refinement of *F*^2^ against ALL reflections. The weighted *R*-factor *wR* and goodness of fit *S* are based on *F*^2^, conventional *R*-factors *R* are based on *F*, with *F* set to zero for negative *F*^2^. The threshold expression of *F*^2^ > σ(*F*^2^) is used only for calculating *R*-factors(gt) *etc*. and is not relevant to the choice of reflections for refinement. *R*-factors based on *F*^2^ are statistically about twice as large as those based on *F*, and *R*- factors based on ALL data will be even larger.

### Fractional atomic coordinates and isotropic or equivalent isotropic displacement parameters (Å^2^)

**Table:**

	*x*	*y*	*z*	*U*_iso_*/*U*_eq_	
C1	0.0401 (3)	0.5352 (3)	0.16636 (17)	0.0418 (6)	
C2	0.0148 (3)	0.3636 (3)	0.15630 (19)	0.0534 (7)	
C3	−0.0178 (4)	0.2719 (4)	0.2248 (3)	0.0758 (11)	
H3	−0.0359	0.1571	0.2205	0.091*	
C4	−0.0243 (5)	0.3456 (5)	0.2992 (2)	0.0844 (12)	
H4	−0.0458	0.2805	0.3440	0.101*	
C5	0.0008 (4)	0.5134 (5)	0.3071 (2)	0.0730 (10)	
H5	−0.0034	0.5631	0.3573	0.088*	
C6	0.0326 (4)	0.6095 (4)	0.24026 (19)	0.0571 (7)	
H6	0.0490	0.7244	0.2452	0.069*	
C7	0.4165 (3)	0.6071 (3)	0.10946 (17)	0.0462 (6)	
C8	0.5725 (3)	0.5282 (5)	0.0766 (2)	0.0738 (10)	
H8A	0.6102	0.5908	0.0310	0.089*	
H8B	0.5489	0.4149	0.0608	0.089*	
H8C	0.6582	0.5283	0.1168	0.089*	
C9	0.0213 (5)	0.2751 (4)	0.0766 (3)	0.0830 (11)	
H9A	0.1276	0.2961	0.0517	0.100*	
H9B	−0.0669	0.3162	0.0429	0.100*	
H9C	0.0075	0.1568	0.0845	0.100*	
N1	0.2818 (3)	0.6051 (3)	0.05903 (12)	0.0413 (5)	
H1N	0.278 (3)	0.556 (3)	0.0138 (12)	0.050*	
O1	−0.0096 (3)	0.6269 (3)	0.01708 (12)	0.0644 (6)	
O2	0.0971 (3)	0.8346 (2)	0.11214 (13)	0.0632 (6)	
O3	0.4067 (2)	0.6678 (3)	0.17544 (11)	0.0588 (5)	
S1	0.09057 (8)	0.66608 (8)	0.08490 (5)	0.04393 (18)	

### Atomic displacement parameters (Å^2^)

**Table:**

	*U*^11^	*U*^22^	*U*^33^	*U*^12^	*U*^13^	*U*^23^
C1	0.0313 (12)	0.0414 (14)	0.0526 (15)	0.0022 (11)	0.0085 (11)	−0.0017 (12)
C2	0.0416 (15)	0.0414 (15)	0.077 (2)	0.0004 (12)	0.0128 (14)	−0.0002 (14)
C3	0.068 (2)	0.0495 (18)	0.110 (3)	0.0058 (16)	0.027 (2)	0.017 (2)
C4	0.078 (2)	0.090 (3)	0.085 (3)	0.011 (2)	0.030 (2)	0.033 (2)
C5	0.069 (2)	0.098 (3)	0.0526 (19)	0.0076 (18)	0.0181 (17)	0.0058 (18)
C6	0.0480 (16)	0.0596 (17)	0.0637 (18)	0.0013 (13)	0.0148 (14)	−0.0082 (16)
C7	0.0392 (14)	0.0478 (14)	0.0515 (17)	−0.0071 (11)	−0.0043 (11)	−0.0001 (13)
C8	0.0401 (15)	0.102 (3)	0.080 (2)	0.0051 (15)	0.0006 (17)	−0.012 (2)
C9	0.084 (2)	0.0521 (18)	0.112 (3)	−0.0123 (16)	0.020 (2)	−0.030 (2)
N1	0.0379 (11)	0.0493 (12)	0.0366 (12)	−0.0012 (9)	0.0021 (9)	−0.0061 (9)
O1	0.0510 (12)	0.0821 (16)	0.0601 (14)	0.0057 (10)	−0.0138 (10)	0.0055 (12)
O2	0.0717 (13)	0.0375 (10)	0.0806 (15)	0.0068 (9)	0.0170 (11)	0.0028 (10)
O3	0.0577 (12)	0.0760 (14)	0.0426 (11)	−0.0075 (10)	−0.0091 (9)	−0.0100 (10)
S1	0.0385 (3)	0.0424 (3)	0.0509 (4)	0.0049 (3)	0.0006 (3)	0.0015 (3)

### Geometric parameters (Å, °)

**Table:**

C1—C6	1.374 (4)	C7—N1	1.367 (3)
C1—C2	1.395 (4)	C7—C8	1.500 (4)
C1—S1	1.765 (3)	C8—H8A	0.9600
C2—C3	1.386 (5)	C8—H8B	0.9600
C2—C9	1.511 (5)	C8—H8C	0.9600
C3—C4	1.378 (5)	C9—H9A	0.9600
C3—H3	0.9300	C9—H9B	0.9600
C4—C5	1.361 (6)	C9—H9C	0.9600
C4—H4	0.9300	N1—S1	1.659 (2)
C5—C6	1.380 (5)	N1—H1N	0.855 (17)
C5—H5	0.9300	O1—S1	1.424 (2)
C6—H6	0.9300	O2—S1	1.4213 (19)
C7—O3	1.209 (3)		
			
C6—C1—C2	121.8 (3)	C7—C8—H8A	109.5
C6—C1—S1	116.8 (2)	C7—C8—H8B	109.5
C2—C1—S1	121.4 (2)	H8A—C8—H8B	109.5
C3—C2—C1	116.5 (3)	C7—C8—H8C	109.5
C3—C2—C9	119.4 (3)	H8A—C8—H8C	109.5
C1—C2—C9	124.1 (3)	H8B—C8—H8C	109.5
C4—C3—C2	122.0 (3)	C2—C9—H9A	109.5
C4—C3—H3	119.0	C2—C9—H9B	109.5
C2—C3—H3	119.0	H9A—C9—H9B	109.5
C5—C4—C3	120.2 (3)	C2—C9—H9C	109.5
C5—C4—H4	119.9	H9A—C9—H9C	109.5
C3—C4—H4	119.9	H9B—C9—H9C	109.5
C4—C5—C6	119.6 (3)	C7—N1—S1	123.94 (18)
C4—C5—H5	120.2	C7—N1—H1N	125.3 (19)
C6—C5—H5	120.2	S1—N1—H1N	109.7 (19)
C1—C6—C5	119.9 (3)	O2—S1—O1	119.00 (13)
C1—C6—H6	120.0	O2—S1—N1	109.12 (11)
C5—C6—H6	120.0	O1—S1—N1	104.10 (12)
O3—C7—N1	121.2 (2)	O2—S1—C1	108.68 (13)
O3—C7—C8	123.9 (3)	O1—S1—C1	110.99 (13)
N1—C7—C8	114.9 (3)	N1—S1—C1	103.79 (11)
			
C6—C1—C2—C3	−0.2 (4)	O3—C7—N1—S1	−6.3 (4)
S1—C1—C2—C3	177.5 (2)	C8—C7—N1—S1	173.3 (2)
C6—C1—C2—C9	180.0 (3)	C7—N1—S1—O2	57.5 (3)
S1—C1—C2—C9	−2.3 (4)	C7—N1—S1—O1	−174.5 (2)
C1—C2—C3—C4	−0.3 (5)	C7—N1—S1—C1	−58.2 (2)
C9—C2—C3—C4	179.6 (3)	C6—C1—S1—O2	−6.2 (3)
C2—C3—C4—C5	0.3 (6)	C2—C1—S1—O2	176.0 (2)
C3—C4—C5—C6	0.1 (5)	C6—C1—S1—O1	−138.9 (2)
C2—C1—C6—C5	0.6 (4)	C2—C1—S1—O1	43.3 (2)
S1—C1—C6—C5	−177.3 (2)	C6—C1—S1—N1	109.8 (2)
C4—C5—C6—C1	−0.5 (5)	C2—C1—S1—N1	−68.0 (2)

### Hydrogen-bond geometry (Å, °)

**Table:**

*D*—H···*A*	*D*—H	H···*A*	*D*···*A*	*D*—H···*A*
N1—H1N···O3^i^	0.86 (2)	1.95 (2)	2.770 (3)	162 (3)

Symmetry codes: (i) −*y*+1, *x*, *z*−1/4.
